# Supplementary material for: Knowledge acquisition is governed by striatal prediction errors
Source: Nat Commun. 2018 Apr 26;9:1673. doi: 10.1038/s41467-018-03992-5 (PMC5919975; doi:10.1038/s41467-018-03992-5)
Supplement: Supplementary file 1 — Supplementary Information [file 41467_2018_3992_MOESM1_ESM.pdf]

Supplementary Information

Knowledge Acquisition is Governed by Striatal Prediction Errors

Pine et al.

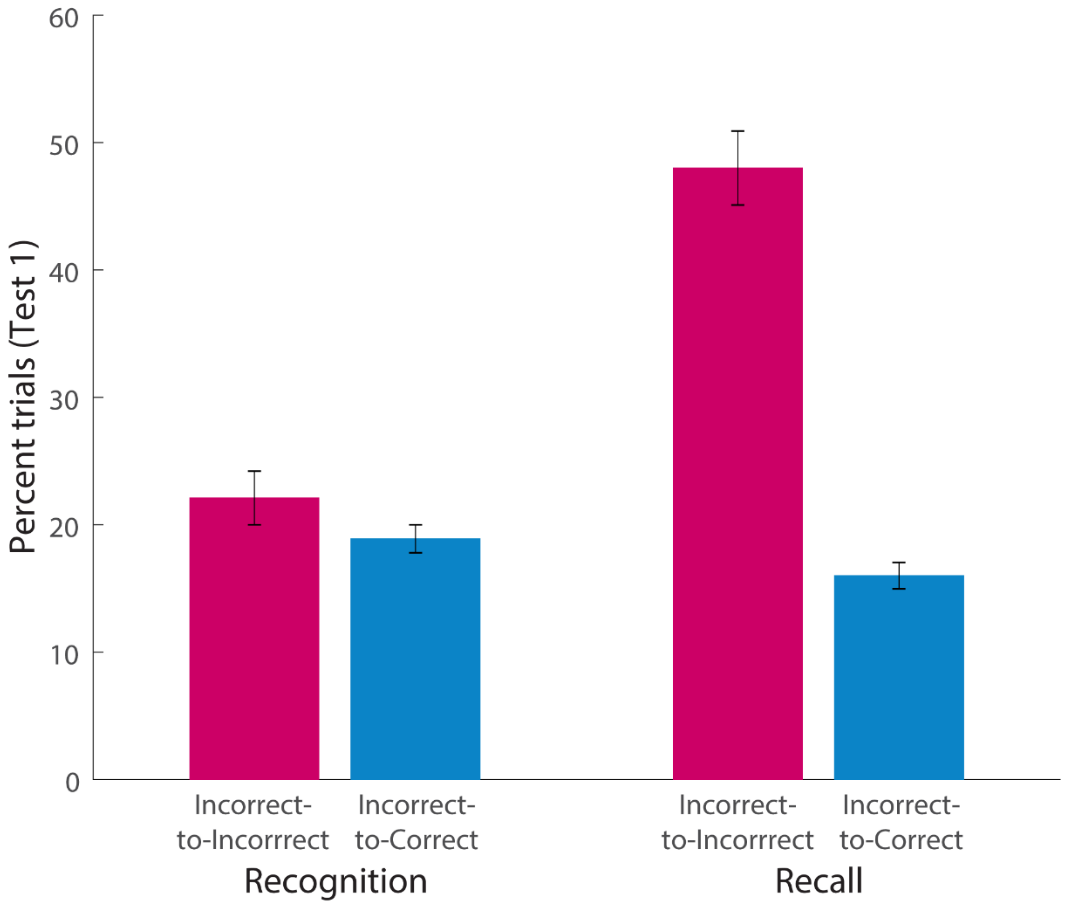

Supplementary Figure 1. Initial accuracy and updating from errors.

Proportion of Test1 trials which were answered incorrectly and were subsequently answered incorrectly (red) or correctly (blue) in Test2. Initial accuracy, and degree of successful updating was inferior in recall relative to recognition (see Results in main text under subheading 'PE magnitude in Test1 correlates with subsequent memory'). Bars depict group mean ± SEM.

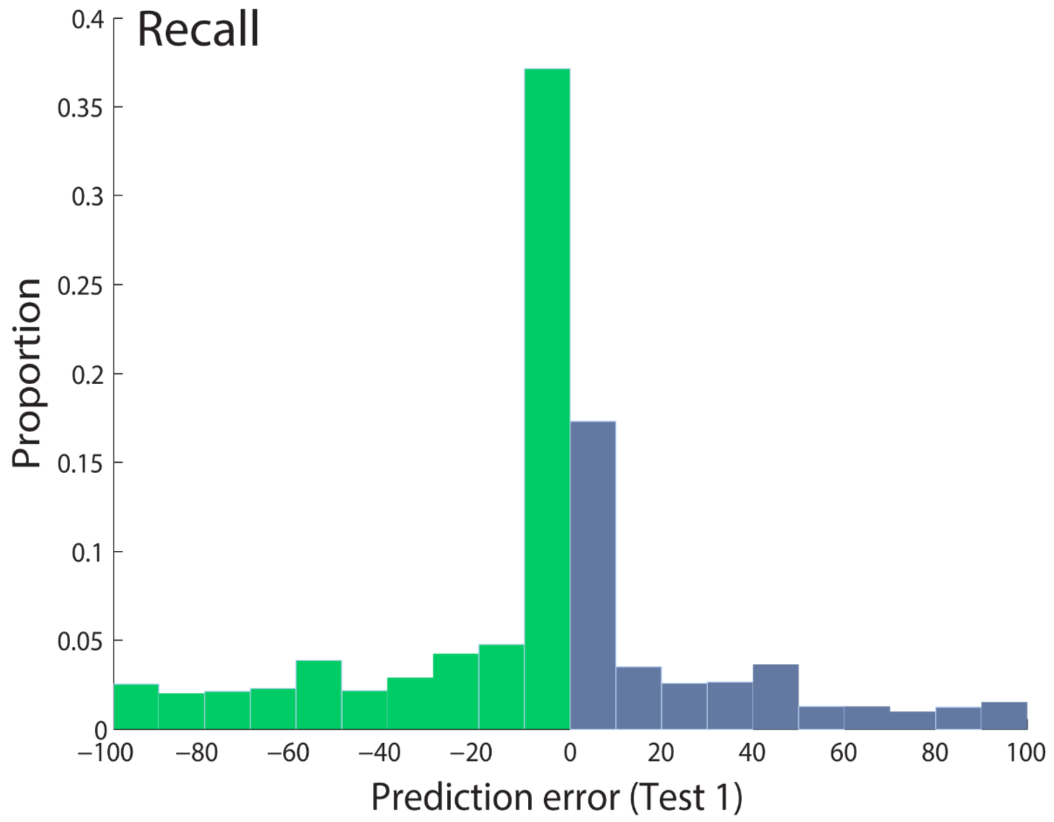

Recall
Proportion
0.4
0.35
0.3
0.25
0.2
0.15
0.1
0.05
0
−100
−80
−60
−40
−20
0
20
40
60
80
100
Prediction error (Test 1)

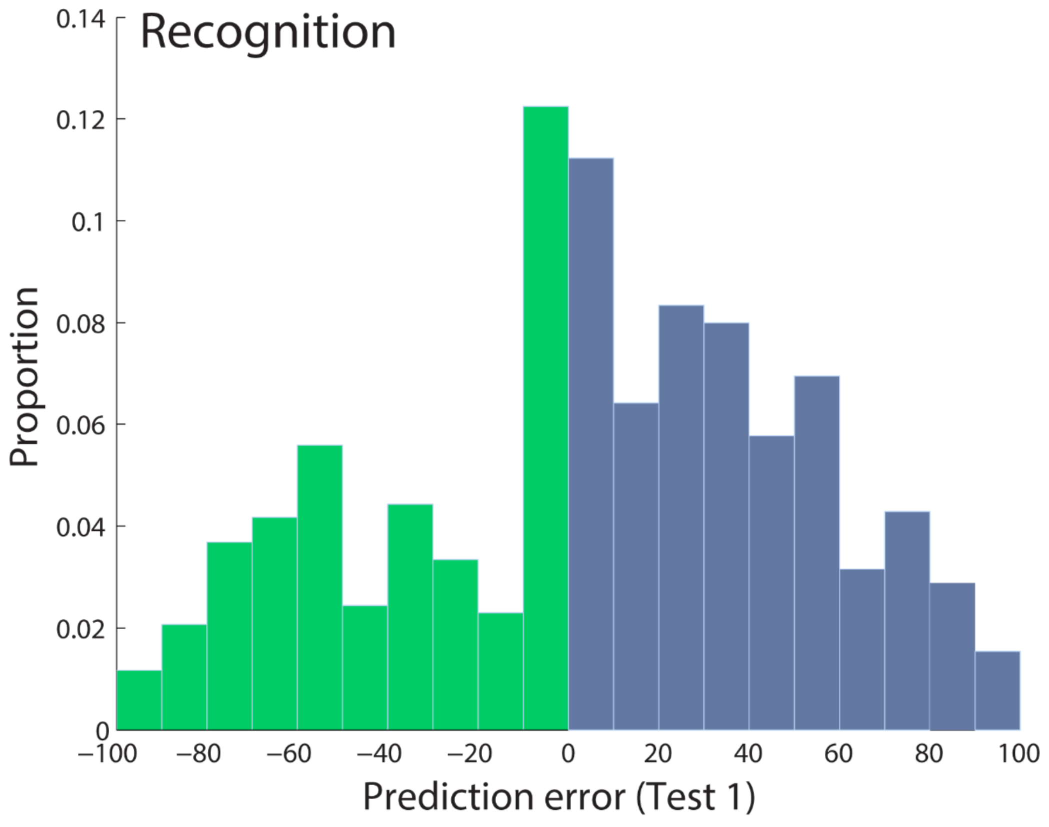

Recognition
Proportion
0.14
0.12
0.1
0.08
0.06
0.04
0.02
0
−100
−80
−60
−40
−20
0
20
40
60
80
100
Prediction error (Test 1)

Supplementary Figure 2. Test1 PE variability and frequency.

Histograms display the relative proportion of PE values occurring in Test1, pooled over all participants, for each study (N=20 in the recall study, N=30 in the recognition study; 100 trials per subject). For incorrect answers (green bars) the PE was calculated as –confidence and for correct answers (blue bars) as 100-confidence.

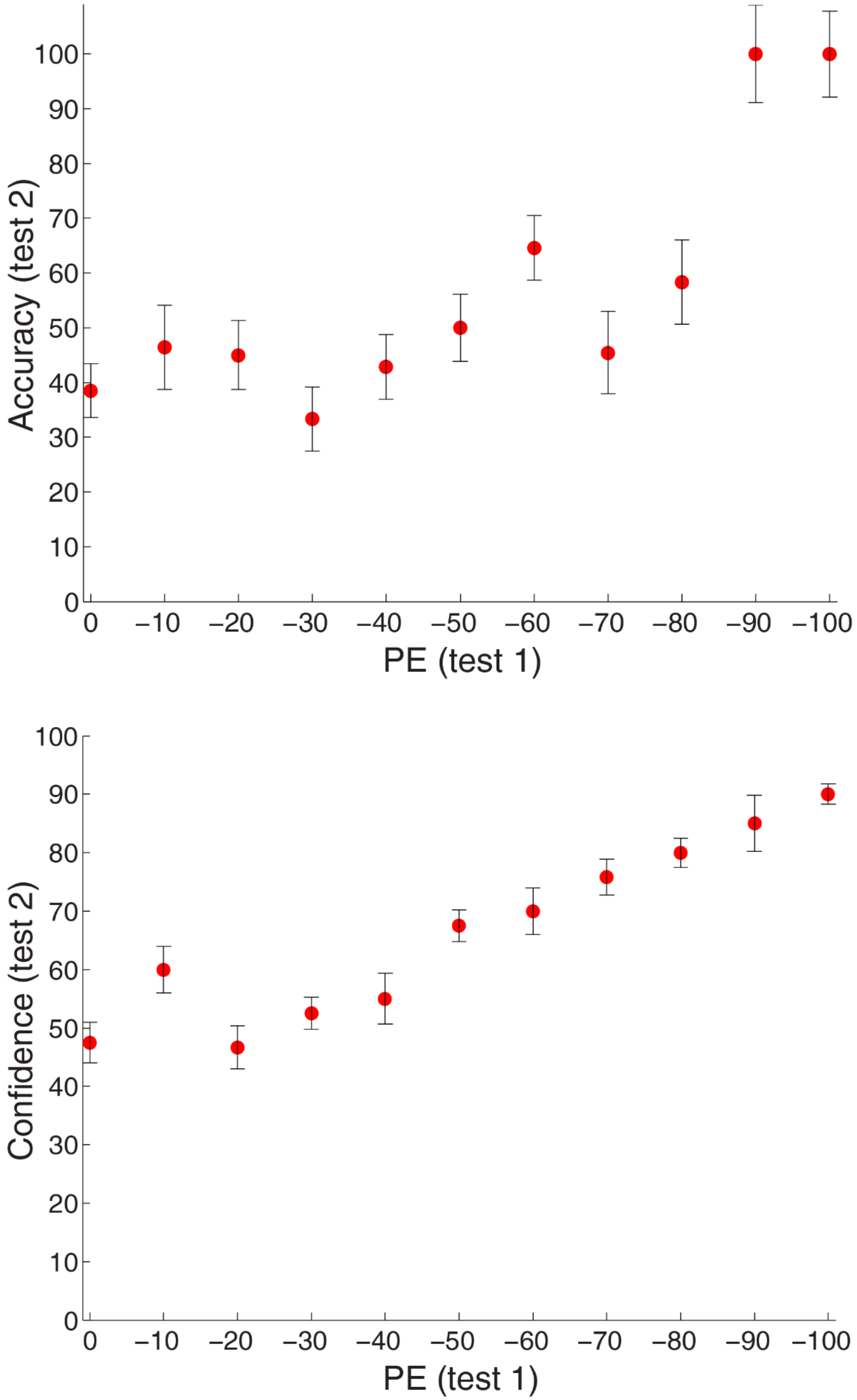

Accuracy (test 2)
100
90
80
70
60
50
40
30
20
10
0
0 −10 −20 −30 −40 −50 −60 −70 −80 −90 −100
PE (test 1)
Confidence (test 2)
100
90
80
70
60
50
40
30
20
10
0
0 −10 −20 −30 −40 −50 −60 −70 −80 −90 −100
PE (test 1)

Supplementary Figure 3. Relationship between PE, subsequent accuracy, and subsequent confidence, for incorrect Test1 trials in the recognition study.

A significant relationship was observed between PE on Test1 for incorrectly answered questions and Test2 accuracy, as well as Test2 confidence for the subset of incorrect-to-correct trials. As PE magnitude increased, efficacy of learning from feedback was superior for both measures (see Results in main text under subheadings 'PE magnitude in Test1 correlates with subsequent memory' and 'Confidence in updated memories correlates with PE'). Displayed are group median values ±SEM.

A

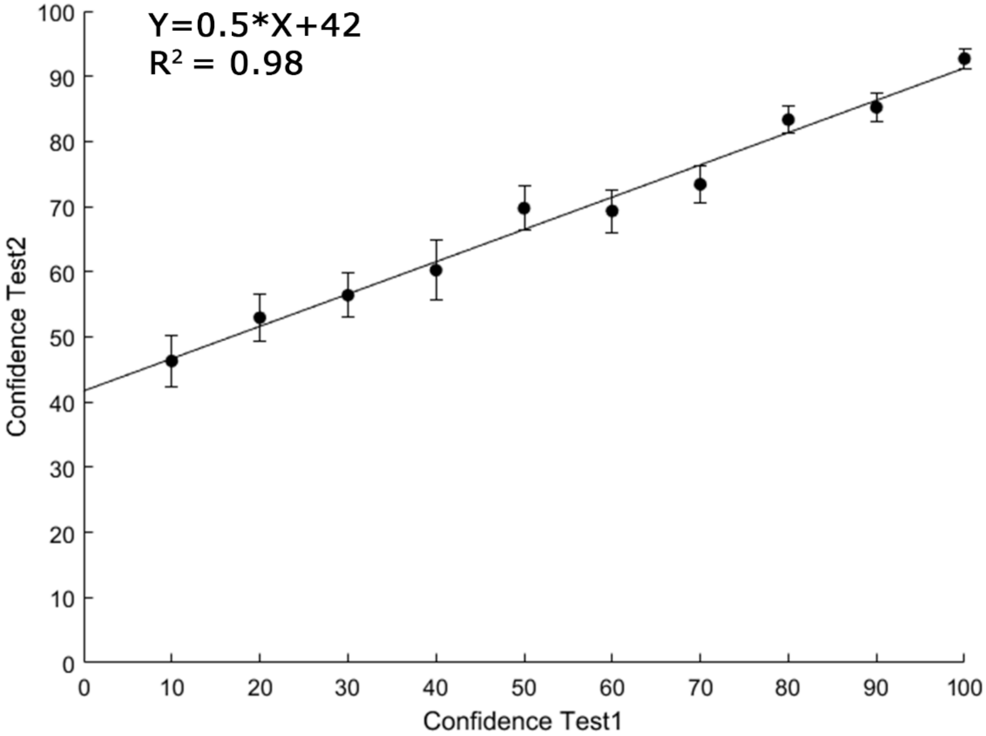

Y=0.5*X+42
R2 = 0.98
100
90
80
70
60
50
40
30
20
10
0
Confidence Test2
0
10
20
30
40
50
60
70
80
90
100
Confidence Test1

B

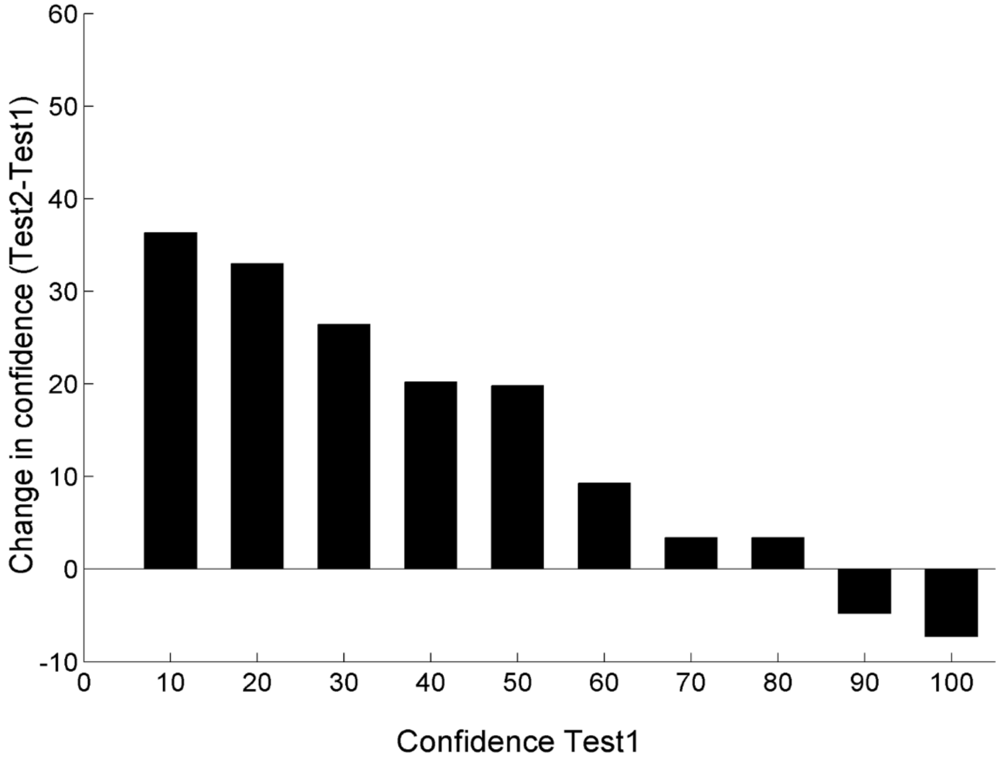

60
50
40
30
20
10
0
-10
Change in confidence (Test2-Test1)
0
10
20
30
40
50
60
70
80
90
100
Confidence Test1

Supplementary Figure 4. Relationship between confidence updating and Test1 PE for trials answered correctly in both tests of the recognition study.

A. A significant linear relationship was observed between PE on Test1 for correctly answered questions (100-confidence) and confidence expressed in the same (correct) answers in Test2. Notably, the slope of this relationship was significantly less than 1, indicating that as Test1 confidence increased (and positive PE decreased from 90 to 0), the degree of confidence updating decreased. Thus, questions answered correctly with low confidence in Test1 (high positive PE) were associated with large increases in confidence in Test2, whereas for higher confidence correct trials (low positive PE), this metric gradually diminished and even became negative, reflecting a decrease in confidence in Test2 (despite having confirmatory feedback in Test1). This result served as a validation of the PE hypothesis, over an alternative hypothesis that the updating effects observed in the incorrect trials could be explained by positing that low confidence results from greater difficulty of learning that information. Note, there were no Test1 trials answered correctly with zero confidence (equivalent to +100 PE). Displayed is group average confidence in Test2 (±SEM) for each confidence level in Test 1, along with the best fitting linear function. **B**. The delta, or change in average confidence from Test1 to Test2.

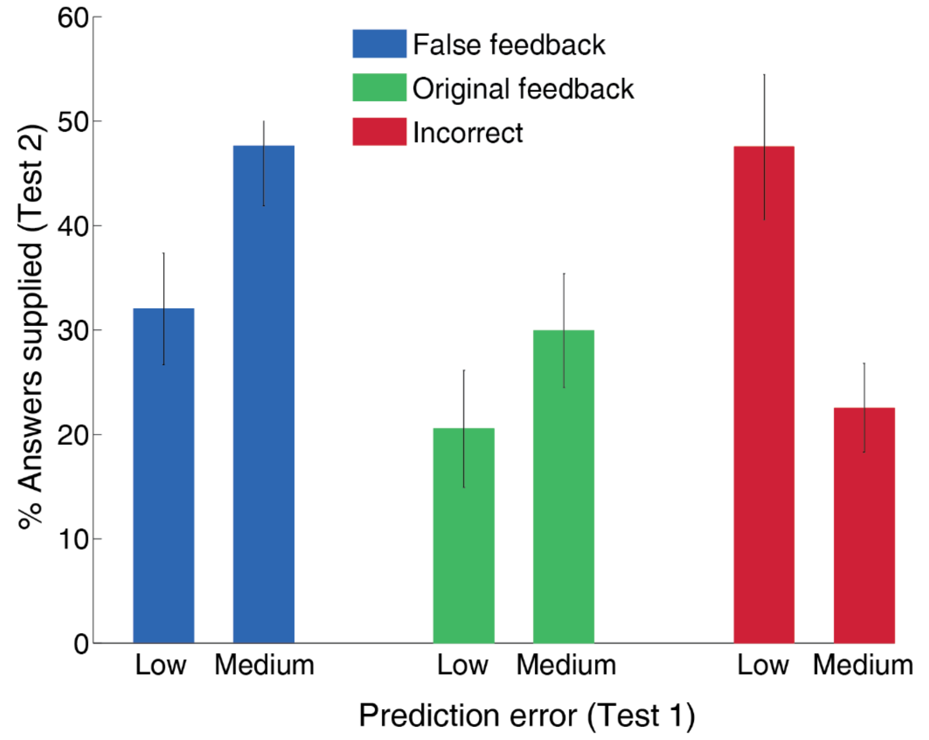
60
50
40
30
20
10
0
% Answers supplied (Test 2)
False feedback
Original feedback
Incorrect
Low
Medium
Low
Medium
Low
Medium
Prediction error (Test 1)

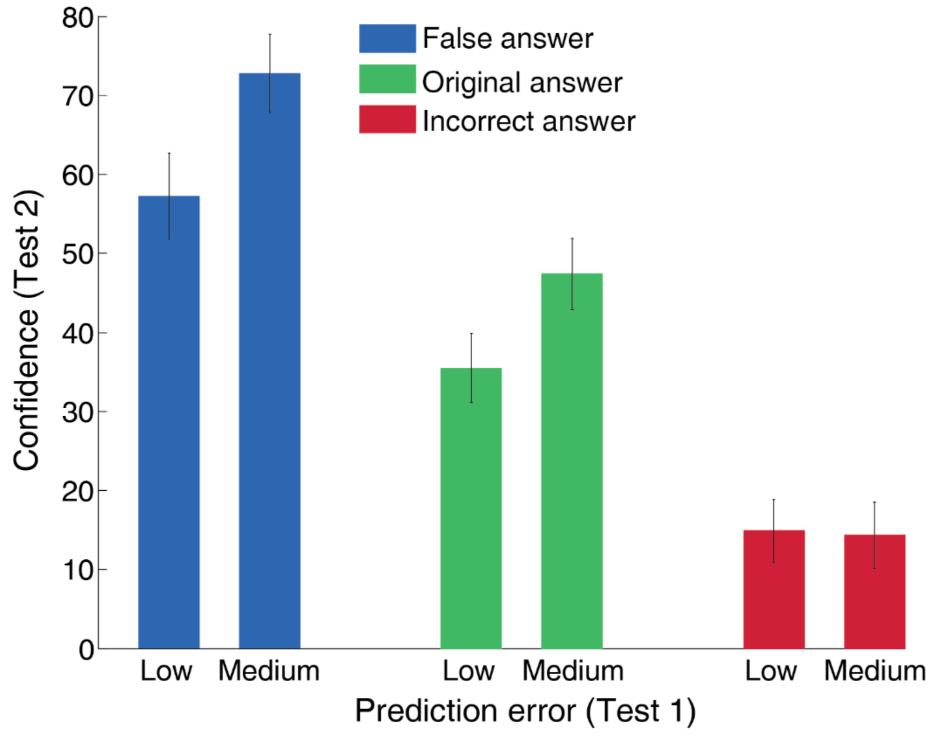
80
70
60
50
40
30
20
10
0
Confidence (Test 2)
False answer
Original answer
Incorrect answer
Low
Medium
Low
Medium
Low
Medium
Prediction error (Test 1)

Supplementary Figure 5. Behavioral summary of memory updating for the subset of Test1 correct, false feedback trials, in the recall study.

There were three possible Test2 answers for this subset of trials: false feedback Test2 answers were instances where the original correct memory was replaced by the false feedback information; original feedback Test2 answers were instances where the correct Test1 answer was again supplied (i.e. memory not overwritten by false feedback); incorrect Test2 answers were instances where no answer was supplied, or an answer was supplied which did not correspond to the original information, or the false feedback. Greater PE induced by false feedback (following medium-confidence correct answers) was associated with an increased likelihood of false feedback recollection in Test2 (above), and – in the subset of correct-to-false feedback trials – with greater confidence in those false memories (below). Depicted are group means ± SEM.

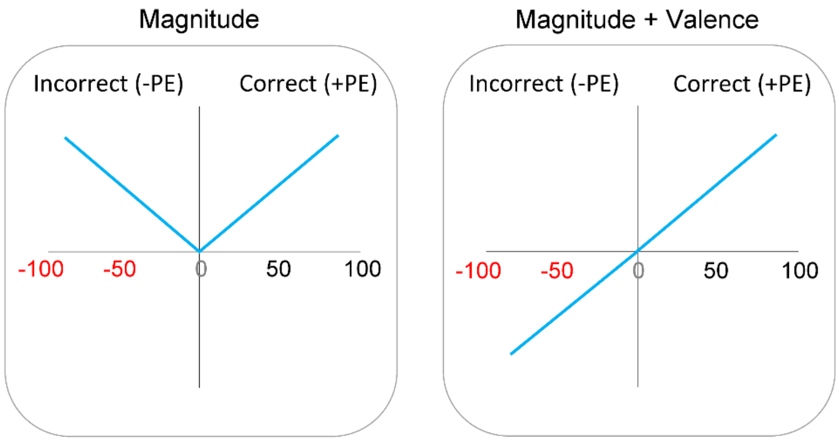

Supplementary Figure 6. Two putative models of PE encoding.

FMRI analyses of PE related neural responses were designed to identify brain regions exhibiting BOLD responses (represented as blue lines) consistent with an encoding of the absolute magnitude (salience), or magnitude and valence of the PE in each trial. The two models can be differentiated by the hypothetical BOLD response to negative PEs (in red), which are expected to be positive in the Magnitude model and negative in the Magnitude + Valence model.

Cluster	X	Y	Z	*t*	N voxels	Location of peak voxel	*p* subsequent accuracy	*p* subsequent confidence
R VS	8	5	-3	4.00	183			0.0053
Cingulate cortex (mid/posterior)	-7	-16	37	3.96	501		0.025	
L VS / caudate nucleus	-10	7	-2	3.94	464	nucleus accumbens		0.0033
L VS	-10	7	-2	3.94	245			0.0006
L caudate nucleus	-17	17	2	3.22	222			0.0453
L VS/VPFC	-17	17	-8	2.43	28			0.017
R DLPFC	46	50	17	3.42	567	middle frontal gyrus		0.0085
R parietal cortex (inferior)	46	-36	33	3.30	405	inferior parietal lobule	0.057	
L DLPFC	-31	39	18	3.19	327	middle frontal gyrus		0.0033
L putamen / claustrum / insula	-31	-5	10	3.18	521	claustrum		
L parietal cortex (inferior)	-58	-40	39	3.17	399	inferior parietal lobule		
Cingulate cortex (mid/anterior)	2	9	38	3.11	224			
Precuneus	-1	-44	44	3.08	125			
R putamen / claustrum/ MTL / insula	29	-8	11	3.02	569	putamen		
R putamen / claustrum (posterior)	29	-8	11	3.02	300	putamen	0.039	
R MTL (anterior)	33	-10	-8	2.74	148	parahippocampal gyrus / amygdala /HC		
R claustrum / insula/ putamen (anterior)	30	5	9	2.73	118	claustrum (anterior)	0.008	
R thalamus	13	-14	0	2.60	27			
L temporal cortex (ventral)	-50	-34	-23	2.60	24	fusiform gyrus		0.0171
L DLPFC	-33	20	48	2.60	87	superior frontal gyrus		
L ventral PFC	-16	57	1	2.58	40	medial frontal gyrus		
R caudate tail	13	-13	19	2.53	37			0.057
R temporal cortex (ventral)	47	-31	-15	2.46	37	fusiform gyrus		
L VLPFC	-25	46	-1	2.38	44			0.011
R PFC (lateral)	49	6	9	2.31	30	precentral gyrus	0.028	
Occipital cortex	-15	-63	29	2.27	170	precuneus		
R caudate nucleus	16	14	19	2.26	15			
L dACC	-9	22	22	2.22	13			0.04
L parietal cortex (superior)	-31	-30	52	2.19	39	postcentral gyrus		
L DLPFC	-21	52	29	2.19	50	superior frontal gyrus		0.036
R temporal cortex	53	-40	10	2.01	32	superior temporal gyrus		
L frontal pole	-21	63	12	1.96	13	superior frontal gyrus		0.004
L parietal cortex (anterior)	-41	-22	44	1.95	16	postcentral gyrus		

Supplementary Table 1. Regions correlating with PE magnitude and valence.

Talairach co-ordinates of the peak voxel, cluster size and *t* values for the regions showing significant correlative activity with signed PE at a threshold of $p < .005$ (see Figure 3 and Results in main text under subheading 'Regions correlating with both valence and magnitude of PE'). The right-hand columns indicate which regions were also predictive of subsequent accuracy and confidence in Test2 (see Figure 5 and Results in main text

under subheading 'PE responsive regions are predictive of subsequent memory'). In light grey are sub clusters / differentiable regions within the large clusters.

Cluster	X	Y	Z	*t*	N voxels	Location of peak voxel
**Positive**						
R LPFC / anterior insula / claustrum	31	22	-5	4.41	671	inferior frontal gyrus
DMPFC	4	30	51	4.15	394	superior frontal gyrus
L LPFC / anterior insula / claustrum	-31	20	-8	3.41	175	inferior frontal gyrus
R DLPFC	42	11	46	3.33	124	middle frontal gyrus
DMPFC	3	38	32	3.07	24	medial frontal gyrus
R DLPFC	46	26	32	2.77	54	middle frontal gyrus
R VLPFC	43	46	-3	2.59	26	middle/inferior frontal gyrus
DMPFC	1	33	40	2.51	12	medial frontal gyrus
**Negative**						
L posterior parahippocampal gyrus / lingual gyrus	-11	-44	2	-4.05	278	parahippocampal gyrus
R dorsal cingulate cortex	13	-19	42	-3.60	41	
R temporal cortex	61	-7	-6	-3.51	39	middle temporal gyrus
L parahippocampal gyrus (mid) / hippocampus	-26	-35	-12	-3.47	100	parahippocampal gyrus
L hippocampus	-29	-27	-7	-2.70	8	
L occcipito-temporo-parietal cortex	-42	-75	23	-3.33	233	angular gyrus
R posterior parahippocampal gyrus / cingulate gyrus	8	-49	8	-3.27	212	parahippocampal gyrus
L DLPFC	-21	20	44	-3.20	52	superior frontal gyrus
R parahippocampal gyrus (mid)	24	-29	-14	-3.18	28	
sgACC	-1	2	-5	-2.96	14	
R parahippocampal gyrus	31	-38	-9	-2.81	14	

Supplementary Table 2. Regions correlating with absolute PE magnitude.

Talairach co-ordinates of the peak voxel, cluster size and *t* values for the regions showing significant correlative activity with unsigned PE at a threshold of $p < .005$ (see Figure 4 and Results in main text under subheading 'Regions correlating with absolute PE magnitude').

Feedback	0	-1 to -33	-33 to -66	-66 to -100
**Original**	15.6	6.3	4.6	9.0
**False**	39.3	14.4	9.6	1.4

Supplementary Table 3. Proportion of erroneous answers according to PE bin and feedback type in the recall study.

Trial type as a percentage of the total number of incorrect answers in Test1. A significant number of false feedback answers were supplied in the recall study to determine false memory adoption following correct responses. Since erroneous answers entailed no/incorrect memory, we did not distinguish between false and original feedback when assessing memory updating, i.e. Test2 answers were scored with respect to the feedback supplied in Test1, not the original material. The updating effect for incorrect Test1 trials was also significant when excluding false-feedback trials from the analysis.
